# Supplementary material for: Relationship between Vitamin Intake and Resilience Based on Sex in Middle-Aged and Older Japanese Adults: Results of the Shika Study
Source: Nutrients. 2022 Nov 27;14(23):5042. doi: 10.3390/nu14235042 (PMC9738285; doi:10.3390/nu14235042)
Supplement: Supplementary file 1 [file nutrients-14-05042-s001.zip › nutrients-1984345-supplementary.pdf]

Supplementary Table S1. Relationship between β-carotene and sex as interaction term on RS

Model	Factors	β	p - value	OR	95%CL	
					Lower	Upper
1	β-carotene*sex	0.000	**< 0.001**	1.000	1.000	1.000
2	β-carotene*sex	0.000	**< 0.001**	1.000	1.000	1.000
3	β-carotene*sex	0.000	**< 0.001**	1.000	1.000	1.000
4	β-carotene*sex	0.000	**< 0.001**	1.000	1.000	1.000

Significant estimates are in bold. Abbreviations: BMI, body mass index; RS, resilience status; OR, odds ratio, CI, confidence interval. Model 1; adjusted for age and BMI, Model 2; adjusted for age, BMI, current smoker, and current drinker. Model 3; adjusted for age, BMI, education, and occupation, Model 4; adjusted for age, BMI, current smoker, current drinker, education, and occupation.

Supplementary Table S2. Relationship between vitamin K and sex as interaction term on RS

Model	Factors	β	p - value	OR	95%CL	
					Lower	Upper
1	Vitamin K*sex	0.002	**0.004**	1.002	1.001	1.004
2	Vitamin K *sex	0.003	**0.003**	1.003	1.001	1.004
3	Vitamin K *sex	0.002	**0.003**	1.002	1.001	1.004
4	Vitamin K *sex	0.003	**0.003**	1.003	1.001	1.004

Significant estimates are in bold. Abbreviations: BMI, body mass index; RS, resilience status; OR, odds ratio, CI, confidence interval. Model 1; adjusted for age and BMI, Model 2; adjusted for age, BMI, current smoker, and current drinker. Model 3; adjusted for age, BMI, education, and occupation, Model 4; adjusted for age, BMI, current smoker, current drinker, education, and occupation.
